# Independent predictors for non-alcoholic fatty liver disease in patients with treatment-naïve chronic hepatitis B

**DOI:** 10.1590/1806-9282.20231036

**Published:** 2024-03-25

**Authors:** Gulsah Tuncer, Ceyda Geyiktepe-Guclu, Osman Faruk Bayramlar, Burcu Atasoy Bozan, Cigdem Yucel, Betul Copur, Kadir Gorkem Guclu, Mustafa Yıldırım, Gonul Sengöz, Filiz Pehlivanoglu

**Affiliations:** 1 Bilecik Training and Research Hospital, Department of Infectious Diseases and Clinical Microbiology – Bilecik, Turkey.; 2 Haseki Training and Research Hospital, Department of Infectious Diseases and Clinical Microbiology – İstanbul, Turkey.; 3 Bakırköy District Health Directorate, Turkish Ministry of Health - Istanbul Health Directorate – İstanbul, Turkey.

**Keywords:** NAFLD, Hepatitis B, BMI, Hyperlipidemia, Diabetes

## Abstract

**OBJECTIVE:**

There are limited data on non-alcoholic fatty liver disease in chronic hepatitis B virus infection. We aimed to determine the predictors for non-alcoholic fatty liver disease in patients with treatment-naïve chronic hepatitis B virus infection.

**METHODS:**

All consecutive treatment-naïve patients with chronic hepatitis B virus infection at the Haseki Training and Research Hospital between October 1, 2021, and September 31, 2022, were retrospectively enrolled. Chronic hepatitis B virus infection is defined by positive serum hepatitis B surface antigen for 6 months or more. Patients with significant alcohol consumption, prolonged steatogenic drug use, malignancy, monogenic hereditary disorders, patients co-infected with hepatitis D virus, hepatitis C virus infection, or human immunodeficiency virus were excluded. Demographic characteristics, anthropometric determinants, laboratory findings, and virological parameters were retrospectively collected from patients’ charts and electronic medical records.

**RESULTS:**

A total of 457 patients with treatment-naïve chronic hepatitis B virus infection were included in the study. The three multivariate regression models revealed that age (p<0.028), body mass index (p=0.046), diabetes mellitus (p=0.030), hemoglobin (p=0.008), platelet (p=0.012), and triglyceride (p=0.002) in Model 1; body mass index (p=0.033), diabetes mellitus (p<0.001), hemoglobin (p=0.008), platelet (p=0.004), LDL (p=0.023), and HDL (p=0.020) in Model 2; and age (p<0.001), body mass index (p=0.033), hemoglobin (p=0.004), platelet (p=0.004), and HDL (p=0.007) in Model 3 were independent predictors.

**CONCLUSION:**

Non-alcoholic fatty liver disease was observed in about one-third of patients with chronic hepatitis B virus infection and was positively associated with older age, higher body mass index, presence of comorbid conditions including diabetes mellitus, increased levels of metabolic laboratory parameters, especially serum triglyceride and LDL, and decreased HDL.

## INTRODUCTION

Both chronic hepatitis B and non-alcoholic fatty liver disease (NAFLD) have caused chronic liver diseases and resulted in poor clinical outcomes^1^. Currently, chronic hepatitis B has affected 296 million patients all around the world^2^. In addition, about a quarter of the global population and one-third of both Western and Asian populations suffer from NAFLD^3-5^. Despite the lower rate of NAFLD in patients with chronic hepatitis B compared with community, NAFLD is still a major public health issue^6,7^. In addition, NAFLD is associated with an increased risk for cardiovascular disease^8^.

Rastogi et al., reported that advanced age, male gender, obesity, lower viral load, and elevated levels of triglycerides, cholesterol, and insulin were associated with hepatic steatosis among patients with chronic hepatitis B virus (HBV). In their study, only serum triglyceride level was detected as an independent predictor for hepatic steatosis^9^. Similarly, Machado et al., showed that male gender, alcohol consumption, body mass index (BMI), obesity, diabetes mellitus, triglycerides, and cholesterol were associated with hepatic steatosis^10^. Although some studies have revealed factors associated with hepatic steatosis in chronic hepatitis B patients, there is still limited data on NAFLD in chronic HBV infection^11-13^. Therefore, in this study, we aimed to determine the predictors for NAFLD in patients with treatment-naïve chronic HBV infection.

## PATIENTS AND METHODS

### Ethical statement

All procedures performed in studies involving human participants were in accordance with the ethical standards of the Declaration of Helsinki. This study was approved by the Ethics Committee of Haseki Training and Research Hospital (approval no: 2022-200, date: November 9, 2022). Written informed consent was waived due to the retrospective nature of this study.

### Study design

All consecutive treatment-naïve patients with chronic HBV infection at the Haseki Training and Research Hospital between October 1, 2021, and September 31, 2022, were retrospectively enrolled. Demographic characteristics (sex, age, and underlying diseases), anthropometric determinants (body mass index), laboratory findings (hemoglobin, platelet, aspartate aminotransferase, alanine aminotransferase, total bilirubin, LDL, HDL, triglyceride, fasting blood glucose, INR, and alpha fetoprotein), and virological parameters (HBV DNA) were retrospectively collected from patients’ charts and electronic medical records. NAFLD was defined as the presence of hepatic steatosis by ultrasonography and the absence of secondary causes of hepatic fat accumulation. The presence of steatosis was evaluated by ultrasonography as grades 1–3.

A total of 472 patients with treatment-naïve patients with chronic HBV infection aged ≥18 years were included. Patients with significant alcohol consumption (n=2, 0.4%), prolonged steatogenic drug use (n=1, 0.2%), malignancy (n=3, 0.6%), monogenic hereditary disorders (n=1, 0.2%), patients co-infected with hepatitis D virus (n=5, 1.1%), hepatitis C virus infection (n=1, 0.21%), or human immunodeficiency virus (n=2, 0.4%) were excluded.

### Definitions

Chronic HBV infection is defined by positive serum hepatitis B surface antigen (HBsAg) for 6 months or more in accordance with AASLD 2018 Hepatitis B Guidance^14^. NAFLD was defined as the presence of hepatic steatosis detected by radiologic imaging or histologic evaluation and the absence of significant alcohol consumption, prolonged use of a steatogenic drug, or other secondary causes of hepatic fat accumulation. Diagnosis criteria of NAFLD were based on NAFLD Practice Guidance from the AASLD^15^. Ultrasonography was used to diagnose NAFLD.

### Statistical analysis

Categorical variables were expressed as frequencies (n) and percentages (%), while numerical variables were expressed as medians (interquartile range). Chi-square and Fisher’s exact tests were used for categorical variables. The Mann-Whitney U test was used for continuous variables. Univariate and multivariate logistic regression analyses were performed to identify independent predictors for NAFLD. A p-value less than 0.05 was considered statistically significant. IBM SPSS Statistics for Windows was used for statistics.

## RESULTS

A total of 457 patients with treatment-naïve chronic HBV infection/hepatitis were included in the study. Of those, 244 (53.4%) were male and the median age was 43 (36–52) years. The median BMI was 26.3 (23.4–29.3). The most common underlying diseases were hypertension (n=75, 16.4%), diabetes mellitus (n=44, 9.6%), hyperlipidemia (n=14, 3.1%), and coronary artery disease (n=12, 2.6%) (Table 1). Twelve (2.6%) patients were HBeAg positive. The median value of HBV DNA was 892 (131–5920) IU/mL (Table 2).

**Table 1:** Comparison of demographic characteristics and underlying diseases in patients with non-alcoholic fatty liver disease and without non-alcoholic fatty liver disease

Parameters		In total		Patients with NAFLD (n=162)		Patients without NAFLD (n=295)		OR	CI	p-value
		n	%	n	%	n	%			
Sex, n (%)	Male	244	53.4	94	58.0	150	50.8	0.748	0.508–1.102	0.141
	Female	213	46.6	68	42.0	145	49.2			
Underlying diseases, n (%)	Yes	127	27.8	64	39.5	63	21.4	2.405	1.579–3.662	**<0.001**
	No	330	72.2	98	60.5	232	78.6			
Diabetes mellitus, n (%)	Yes	44	9.6	26	16	18	6.1	2.942	1.559–5.552	**0.001**
	No	413	90.4	136	84	256	93.9			
Hypertension, n (%)	Yes	75	16.4	36	22.2	39	13.2	1.875	1.137–3.094	**0.013**
	No	382	83.6	126	77.8	256	86.8			
Chronic artery diseases, n (%)	Yes	12	2.6	7	4.3	5	1.7	2.636	0.823–8.445	0.091
	No	444	97.4	154	95.7	290	98.3			
Chronic kidney disease, n (%)	Yes	8	1.8	2	1.2	6	2	0.602	0.120–3.018	0.533
	No	449	98.2	160	98.8	289	98			
Chronic obstructive pulmonary disease, n (%)	Yes	7	1.5	3	1.9	4	1.4	1.373	0.303–6.210	0.702
	No	450	98.5	159	98.1	291	98.6			
Neurological disease, n (%)	Yes	8	1.8	2	1.2	6	2	0.602	0.120–3.018	0.533
	No	449	98.2	160	98.8	289	98			
Hyperlipidemia, n (%)	Yes	14	3.1	9	5.6	5	1.7	3.412	1.124–10.359	**0.022**
	No	443	96.9	153	94.4	290	98.3			
HBeAg positive, n (%)	Yes	12	2.6	4	2.5	8	2.7	0.908	0.269–3.064	0.877
	No	445	97.4	158	97.5	287	97.3			

Statistically significant values are indicated in bold.

**Table 2:** Comparison of age, body mass index, viral load, laboratory parameters, and liver histopathology scores in patients with non-alcoholic fatty liver disease and without non-alcoholic fatty liver disease

Parameters	In total	Patients with NAFLD (n=162)	Patients without NAFLD (n=295)	p-value
	Median (IQR 25–75)	Median (IQR 25–75)	Median (IQR 25–75)	
Age, years, median (IQR)	43 (36–52)	47 (40–55)	42 (34–51)	**<0.001**
BMI, median (IQR)	26.3 (23.4–29.3)	28.1 (25.8–31.5)	25.5 (22.9–28.5)	**<0.001**
HBV-DNA, IU/mL, median (IQR)	892 (131–5920)	572 (88–3730)	1030 (187–7200)	**0.021**
Hemoglobin, g/dL, median (IQR)	14 (13–15.3)	14.6 (13.2–15.4)	14 (12.6–15.3)	**0.014**
Platelet /mm^3^, median (IQR)	232 (196–269)	237 (204–278)	227 (194–259)	**0.017**
Aspartate aminotransferase (AST), IU/mL, median (IQR)	20 (17–24)	20 (17–25)	20 (17–24)	0.607
Alanine aminotransferase (ALT), IU/mL median (IQR)	20 (15–29)	20 (15–32)	19 (15–28)	0.074
Total bilirubin, mg/dL, median (IQR)	0.44 (0.33–0.64)	0.44 (0.34–0.61)	0.46 (0.33–0.65)	0.538
LDL, mg/dL, median (IQR)	103 (84–128)	110 (86–136)	100 (82–122)	**0.003**
HDL, mg/dL, median (IQR)	47 (40–56)	45 (36–52)	49 (41–58)	**<0.001**
Triglyceride, mg/dL, median (IQR)	109 (77–160)	148 (104–193)	95 (71–135)	**<0.001**
Fasting blood glucose, mg/dL, median (IQR)	94 (88–105)	99 (89–110)	93 (87–103)	**0.003**
INR, median (IQR)	1.0 (1.0–1.1)	1.0 (1.0–1.0)	1.0 (1.0–1.1)	**0.004**
Alfa fetoprotein, ng/mL, median (IQR)	2.5 (1.8–3.6)	2.5 (1.8–3.4)	2.6 (1.8–3.6)	0.78
FIB-4 score, median (IQR)	0.8 (0.7–1.2)	0.9 (0.7–1.1)	0.8 (0.6–1.2)	0.967
Fibrosis, median (IQR)	1 (0–1)	0 (0–1)	1 (0–1)	0.647
Hepatic activity index (HAI), median (IQR)	4 (3–5)	4 (3–5)	4 (3–6)	0.636

Statistically significant values are indicated in bold.

Non-alcoholic fatty liver disease was observed in 162 (35.4%) patients. Patients with NAFLD were older than patients without NAFLD (47 years vs. 42 years, p<0.001). Presence of underlying diseases (at least one or more) (39.5% vs. 21.4%, p<0.001), diabetes mellitus (16% vs. 6.1%, p=0.001), hypertension (22.2% vs. 13.2%, p=0.013), and hyperlipidemia (5.6% vs. 1.7%, p=0.022) were more common in patients with NAFLD than without NAFLD (Table 1).

The median values of HBV DNA (p=0.021) and HDL levels (p<0.001) were lower in patients with NAFLD than those without NAFLD. However, BMI (p<0.001), hemoglobin (p=0.014), platelet count, LDL (110 mg/dL vs. 100 mg/dL, p=0.003), HDL (p<0.001), triglyceride (p<0.001), fasting blood glucose (p=0.003), and INR (p=0.004) were higher in patients with NAFLD (Table 2).

In univariate analysis, age (p<0.001), BMI (p=0.001), hypertension (p=0.014), diabetes mellitus (p=0.001), hyperlipidemia (p<0.030), hemoglobin (p=0.009), platelet (p=0.032), LDL (p=0.001), HDL (p<0.001), and triglyceride (p<0.001) were predictors for NAFLD in patients with chronic hepatitis B (Table 3).

**Table 3:** Univariate and multivariate analyses for predicting non-alcoholic fatty liver disease in patients with chronic hepatitis B

Parameters	Univariate analysis			Multivariate Model 1			Multivariate Model 2			Multivariate Model 3		
	OR	CI	p	OR	CI	p	OR	CI	p	OR	CI	p
Age	1.029	1.013–1.046	**<0.001**	1.034	1.004–1.065	**0.028**	–	–	–	1.048	1.023–1.074	**<0.001**
Body mass index	1.072	1.028–1.117	**0.001**	1.049	1.001–1.100	**0.046**	1.051	1.004–1.101	**0.033**	1.048	1.004–1.095	**0.033**
Hypertension	1.875	1.137–3.094	**0.014**	1.009	0.425–2.394	0.983	–	–	–	–	–	–
Diabetes mellitus	2.942	1.559–5.552	**0.001**	3.446	1.130–10.515	**0.030**	5.711	2.075–15.722	**<0.001**	–	–	–
Hyperlipidemia	3.412	1.124–10.359	**0.030**	1.248	0.241–6.470	0.792	–	–	–	–	–	–
Hemoglobin	1.165	1.039–1.306	**0.009**	1.315	1.076–1.608	**0.008**	1.292	1.068–1.564	**0.008**	1.336	1.098–1.625	**0.004**
Platelet count	1.004	1.000–1.007	**0.032**	1.008	1.002–1.014	**0.012**	1.008	1.003–1.014	**0.004**	1.009	1.003–1.015	**0.004**
Triglyceride	1.011	1.007–1.014	**<0.001**	1.009	1.003–1.014	**0.002**	–	–	–	–	–	–
LDL	1.010	1.004–1.016	**0.001**	1.008	0.998–1.017	0.130	1.010	1.001–1.019	**0.023**	–	–	–
HDL	0.967	0.952–0.983	**<0.001**	0.987	0.957–1.017	0.384	0.969	0.943–0.995	**0.020**	0.963	0.937–0.990	**0.007**

Model 1: All significant variables in univariate analysis were included. Model 2: BMI, diabetes mellitus, hemoglobin, platelet, and LDL were included. Model 3: Age, BMI, hemoglobin, platelet, and HDL were included. Statistically significant values are indicated in bold.

The three multivariate regression models revealed that age (p<0.028), BMI (p=0.046), diabetes mellitus (p=0.030), hemoglobin (p=0.008), platelet (p=0.012), and triglyceride (p=0.002) in Model 1; BMI (p=0.033), diabetes mellitus (p<0.001), hemoglobin (p=0.008), platelet (p=0.004), LDL (p=0.023), and HDL (p=0.020) in Model 2; and age (p<0.001), BMI (p=0.033), hemoglobin (p=0.004), platelet (p=0.004), and HDL (p=0.007) in Model 3 were independent predictors (Table 3).

## DISCUSSION

In this study, the prevalence of NAFLD among patients with treatment-naïve chronic HBV infection was 35.4% (n=162). We found that age, BMI, diabetes mellitus, hemoglobin, serum triglyceride, LDL, and HDL were independent predictors for NAFLD.

Non-alcoholic fatty liver disease is commonly associated with obesity, diabetes mellitus, and elevated cholesterol^8^. In the study of Zhu et al., obesity and diabetes mellitus were associated with 8.5-fold and 2-fold increased risk for NAFLD among patients with chronic hepatitis B, respectively^16^. In this study, we observed a 2–3.5-fold increased risk for NAFLD in patients with hypertension, diabetes mellitus, and hyperlipidemia. Furthermore, the presence of diabetes mellitus was independently associated with about 3.5-fold increased risk for NAFLD among patients with treatment-naïve chronic HBV infection in multivariate regression analysis.

The association between HBV replication and hepatic steatosis is also unclear^17^. While some studies demonstrated that there is a negative association between hepatic steatosis and HBV DNA^18^, others have reported no associations between viral load and hepatic steatosis^19^. In a recent study, Wang et al., demonstrated that HBV DNA level was negatively and independently associated with NAFLD in the pediatric population with chronic hepatitis B^20^. Similar to our study, Zhu et al., reported that viral load or other viral factors were not independently associated with NAFLD^16^. Similarly, the negative association between NAFLD and HBV seromarkers was also supported by studies in animal models. In one animal model of NAFLD-CHB comorbidity, HBeAg, HBsAg, hepatitis B core antigen, and HBV DNA levels were higher in mice without NAFLD than those with NAFLD, although the mechanism was not explored^21^. In our study, a significant association between HBV DNA and NAFLD was not detected. This could be because the majority of our study group consisted of grade-1 steatosis and the rate of advanced steatosis was low.

Minakari et al., evaluated 132 treatment-naïve patients. Of those, 35 (26.5%) were HBeAg positive and 56 (42.4%) had NAFLD^12^. In univariate analysis, patients without steatosis were significantly older than those with steatosis. HBV DNA levels were lower in those with steatosis, but no statistically significant difference was found. BMI, serum triglyceride, fasting blood glucose, and GGT were found as predictors for NAFLD in univariate analysis. However, only serum triglyceride was an independent predictor in multivariate analysis. In the study of Yun et al., among untreated young males with chronic hepatitis B, serum insulin, total cholesterol, and triglyceride were significantly higher in patients with steatosis than in patients without steatosis^22^. The researchers reported that homeostatic model assessment for insulin resistance and triglyceride was found to be significant in the multivariate analysis. In a study conducted by Vigano et al., the severity of steatosis was significantly associated with advanced age, male gender, and higher BMI^23^. In their study, a higher prevalence of hyperglycemia was observed in patients with mild steatosis, while triglyceride levels increased progressively with the severity of steatosis. Nau et al., included 83 patients with an HbeAg-positive rate of 9.1%^24^. Fatty liver was observed in 11.3% of patients. They reported that total cholesterol was higher and prothrombin time was longer in patients with steatosis on ultrasound. Higher fasting insulin levels and higher BMI were found in patients with steatosis. AST levels were lower in patients with steatosis.

Our study had several strengths. First, the sample size was relatively high. Second, we could add various variables in the multivariate regression models. This study had some limitations. First, this study was conducted in a single center. Second, we used ultrasonography to identify NAFLD. Histopathological examination was not evaluated. Third, because the prevalence of patients with grade-3 steatosis in our study group was rare, this might affect the generalizability of our results. Therefore, large-scale studies are needed to identify associated factors for NAFLD in patients with advanced hepatic steatosis.

## CONCLUSION

Non-alcoholic fatty liver disease was observed in about one-third of patients with chronic HBV infection and was positively associated with older age, higher BMI, presence of comorbid conditions including diabetes mellitus, increased levels of metabolic laboratory parameters, especially serum triglyceride and LDL, and decreased HDL. However, neither HBV DNA levels nor HBeAg positivity were independent predictors for NAFLD.
